# A cognitive pathway to punishment insensitivity

**DOI:** 10.1073/pnas.2221634120

**Published:** 2023-04-03

**Authors:** Philip Jean-Richard-dit-Bressel, Jessica C. Lee, Shi Xian Liew, Gabrielle Weidemann, Peter F. Lovibond, Gavan P. McNally

**Affiliations:** ^a^School of Psychology, University of New South Wales, Sydney 2052, NSW, Australia; ^b^School of Psychology, Western Sydney University Locked Bag 1797, Penrith NSW 2751, Australia

**Keywords:** punishment, compulsivity, individual differences

## Abstract

Individuals differ in their sensitivity to the adverse consequences of their actions, leading some to persist in maladaptive behaviors. Two pathways have been identified for this insensitivity: a motivational pathway based on excessive reward valuation and a behavioral pathway based on autonomous stimulus–response mechanisms. Here, we identify a third, cognitive pathway based on differences in punishment knowledge and use of that knowledge to suppress behavior. We show that distinct phenotypes of punishment sensitivity emerge from differences in what people learn about their actions. Exposed to identical punishment contingencies, some people (sensitive phenotype) form correct causal beliefs that they use to guide their behavior, successfully obtaining rewards and avoiding punishment, whereas others form incorrect but internally coherent causal beliefs that lead them to earn punishment they do not like. Incorrect causal beliefs were not inherently problematic because we show that many individuals benefit from information about why they are being punished, revaluing their actions and changing their behavior to avoid further punishment (unaware phenotype). However, one condition where incorrect causal beliefs were problematic was when punishment is infrequent. Under this condition, more individuals show punishment insensitivity and detrimental patterns of behavior that resist experience and information-driven updating, even when punishment is severe (compulsive phenotype). For these individuals, rare punishment acted as a “trap,” inoculating maladaptive behavioral preferences against cognitive and behavioral updating.

## Significance

Insensitivity to the adverse consequences of our actions drives problematic behaviors such as those observed in substance use disorders, conduct disorder, and antisocial personality disorder. Two pathways have been proposed for this insensitivity: a motivational pathway based on differences in reward valuation and a behavioral pathway based on autonomous stimulus–response mechanisms. Here, we identify a third, cognitive pathway based on differences in awareness of the adverse consequences of one’s actions. We show that when the costs of actions are rare, learning via experience and information does not always yield veridical causal knowledge or optimum decision-making, causing some individuals to continually incur punishments that they neither like nor want.

Punishment learning is central to decision-making and assessment of risk. When successful, this learning maximizes probability of our survival by reducing behaviors that cause us harm and sustaining mutually beneficial behaviors essential for group cooperation and social cohesion (1–3). However, punishment learning is not always successful. Some people readily learn to reduce behaviors that have adverse consequences, whereas others do not (4, 5). Insensitivity to the adverse consequences of our actions drives decision-making deficits and problematic, compulsive behaviors, including substance use disorders (6), antisocial personality disorder and conduct disorder, and oppositional defiant disorder in children, (7, 8), and contributes to high rates of recidivism in these populations (9).

At least two pathways contribute to these decision-making deficits: a motivational pathway characterized by value distortions that skew the expected utility functions governing action selection (10–15) and a behavioral pathway characterized by dominance of autonomous stimulus–response mechanisms (16–18). However, neither of these pathways can explain why individuals who persist in maladaptive behaviors often fail to recognize relationships between their actions and adverse consequences (19–23).

We recently showed that punishment insensitivity readily emerges from the different beliefs people hold about their actions (4). Punishment-sensitive individuals acquired correct punishment contingency knowledge that they used to reduce punished actions. In contrast, punishment-insensitive individuals failed to develop accurate punishment contingency beliefs. Despite disliking punishment, insensitive individuals cannot withhold detrimental behavior because their understanding about the causes of punishment is wrong.

Although a lack of awareness about the consequences of one’s actions can cause enduring patterns of detrimental behavior, lack of awareness is not inherently problematic and may be quite common (24). We learn in different ways (e.g., personal experience, observation, instruction) and readily integrate knowledge and evidence from different modalities to improve our understanding. Failing to correctly learn about punishment from one source (e.g., experience) does not mean that behavior is fundamentally resistant to change. Moreover, punishment contingencies vary in their visibility to the individual. Detection may sometimes be difficult because punishment is delayed relative to, and imperfectly correlated with, the action that earned it (25), but this should be overcome by improving visibility of the punishment contingency.

Across three experiments, we show how differences in awareness of the relationship between one’s actions and punishers can function as a third, cognitive pathway to punishment decision-making deficits. We imposed punishment contingencies of varying visibility on participants seeking financial reward. We then provided information about the sources of punishment before providing further opportunity to seek reward under risk of punishment. We show that explicit information is a potent means of addressing this lack of awareness, rescuing most but not all people from continued self-inflicted detriment. However, this intervention was relatively ineffective when punishment was rare. When punishment was rare, learning via experience or information did not readily yield veridical causal knowledge or optimum decision-making, even when those costs were severe.

## Results

### Experiment 1: From Punishment Insensitive to Compulsive.

**Fig. 1.**
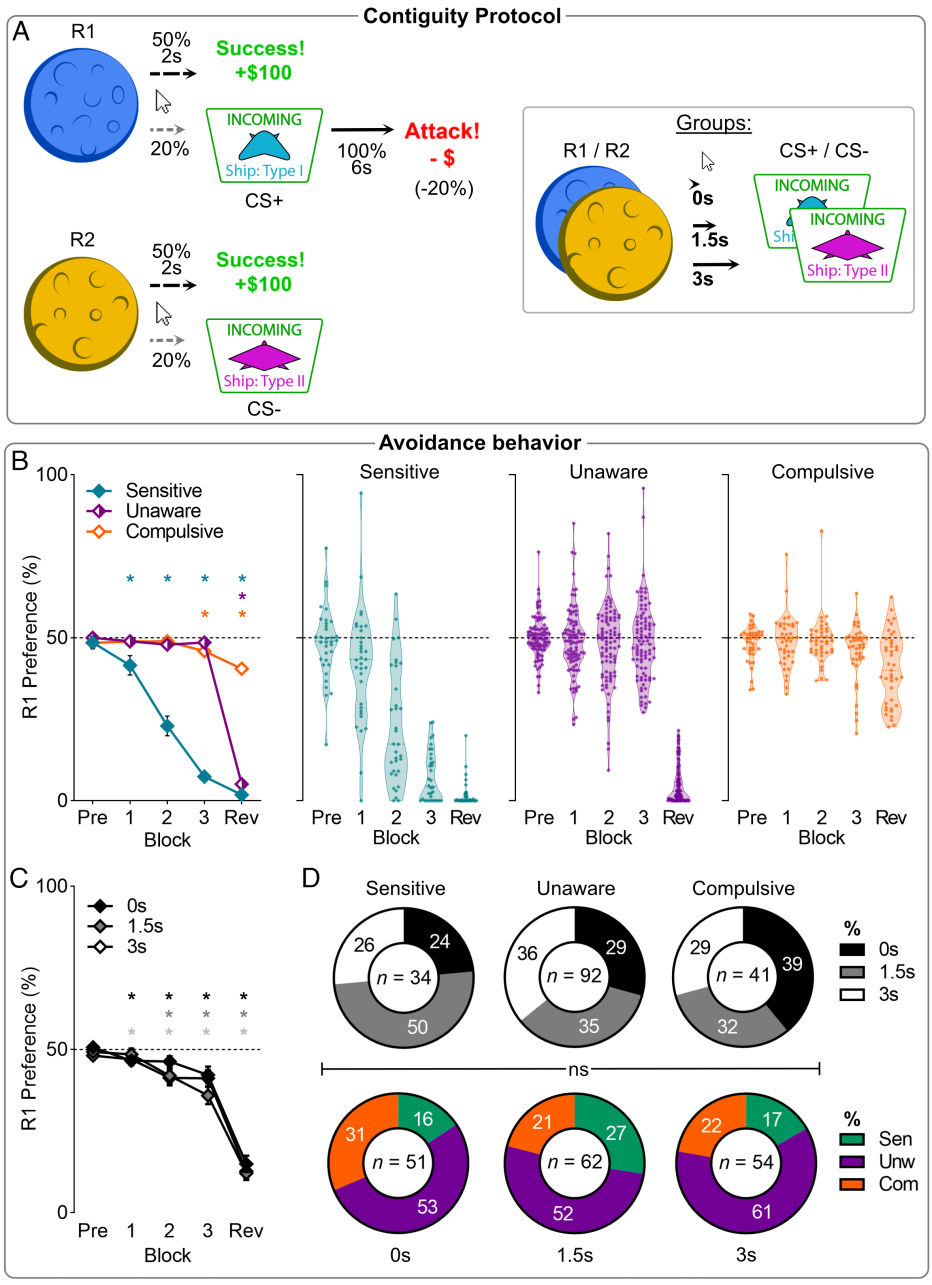
Phenotypes of punishment avoidance across Response-CS delays in experiment 1. (*A*) Conditioned punishment task. (*B*) Mean (±SEM) punished action preferences for the 3 behavioral phenotypes. Pre = pre-punishment; Rev = post-reveal. (*C*) Mean (±SEM) punished action (R1) preference across blocks per contiguity group. Contiguity manipulations did not significantly affect avoidance. (*D*) Composition of clusters by contiguity groups (*Top*), and vice versa (*Bottom*). Contiguity group did not significantly determine cluster phenotype. *P < 0.05 single mean *t* test vs. 0.5 preference.

A total of 167 participants [123 identifying as female, 1 as other, 17 to 32 y old (*M* = 19.17)] underwent the “Planets and Pirates” task (Fig. 1*A*) (4). In the first phase (pre-punishment), participants made mouse click responses (R1 and R2) on two continuously presented planets to earn points. These responses were reinforced with 50% probability. There were two, 3-min blocks of pre-punishment training. Under these conditions, all participants learned to accumulate points, with R1 and R2 occurring at similar rates across these blocks (*SI Appendix*, Fig. S1*A*).

In the second punishment phase, participants received three blocks of training (Fig. 1*A*). Reward contingencies were identical to the first phase, but a conditioned punishment contingency was now introduced. Under this contingency, R1 (punished action) yielded a 6-s on-screen presentation of a spaceship (CS+) with 20% probability. This was followed by an “attack” whereby participants lost 20% of their accumulated points. By contrast, R2 (unpunished action) yielded a different spaceship (CS−) with 20% probability, but this did not cause points loss. Learning is shown by a reduction in the punished (R1) relative to unpunished action (R2). To vary punishment visibility (25), participants were randomly allocated to one of three groups that differed in the delay between response and CS presentation [0s (n = 51), 1.5s (n = 62), or 3s (n = 54)].

At the end of the second phase, participants were presented with on-screen information explicitly revealing the punishment contingencies they were receiving (R1→CS+→Attack; R2→CS−→nothing). Understanding was assessed using an on-screen knowledge test that participants were required to answer correctly before proceeding to a final block of post-reveal punishment trials with the same punishment contingencies.

Overall, participants were sensitive to punishment, reducing punished relative to unpunished actions across pre-reveal punishment blocks [F(1,166) = 40.73, *P* < 0.001; Fig. 1*C*] which continued following reveal [F(1,166) = 225.21, *P* < 0.001]. This learning did not differ according to contiguity group [group: F(2,164) = 0.980, *P* = 0.377; block*group pre-reveal: F(2,164) = 1.773, *P* = 0.173; block*group post-reveal: F(2,164) = 0.652, *P* = 0.522; Fig. 1*C*]. Information about the correct contingencies improved punishment performance in the post-reveal block, further increasing preference for the safe action over the punished action [t(166) = −27.64, *P* < 0.001]. These results demonstrate successful punishment learning and the ability of explicit information about correct contingencies to improve this learning.

Nonetheless, there was pronounced variation between individuals in punishment learning and the impact of information. A TwoStep clustering algorithm (26) using the last two punishment blocks (3 and Rev) identified 3 clusters (Fig. 1*B*): a “sensitive” cluster (n = 34) that acquired pronounced avoidance prior to the contingency reveal, an “unaware” cluster (n = 92) that failed to acquire avoidance before the reveal but showed pronounced avoidance following the reveal, and finally, a “compulsive” cluster (n = 41) that did not avoid the punished response, even after contingencies were fully revealed to them. It is important to note that we use the term compulsive to refer to this persistence of behavior in the face of punishment without connotation of the underlying causes for this pattern of behavior.

To assess how these clusters did vs. did not differ from each other, we assessed behavioral preferences across blocks. Clusters did not differ in pre-punishment response preference [F(2,164) = 0.92, *P* = 0.401], but did during pre-reveal punishment [P1: F(2,164) = 5.492, *P* = 0.005; P2: F(2,164) = 54.37, *P* < 0.001; P3: F(2,166) = 206.4, *P* < 0.001]. Only the Sensitive cluster showed a significant change in preference across pre-reveal blocks [F(1, 33) = 314.77, *P* < 0.001]. The impact of reveal [F(1,164) = 287.07, *P* < 0.001] depended on cluster [block*cluster: F(2,164) = 186.93, *P* < 0.001]. Unaware participants showed the most pronounced change in preference [block (Unaware): F(1,91) = 951.1, *P* < 0.001], but all clusters showed a significant change [block (Sensitive): F(1, 33) = 13.60, *P* < 0.001; block (Compulsive): F(1, 40) = 6.853, *P* = 0.012]. After reveal, there was no significant difference between Unawares and Sensitives (*P* = 0.073), but these clusters showed better punishment avoidance than Compulsives (*P* < 0.001). The visibility of punishment as manipulated by action–punishment contiguity did not affect cluster allocation as there was no effect of delay group on avoidance phenotype [χ^2^(4) = 4.371, *P* = 0.358] (Fig. 1*D*). Sex of the participant was also not a significant factor on avoidance phenotype [χ^2^(4) = 3.367, *P* = 0.498].

These behavioral differences were consequential. Pre-reveal, Sensitives gained the most and Unawares the least points (*SI Appendix*, Fig. S1*B*). After reveal, Unawares gained as many points as Sensitives but Compulsives gained the least. So, Unawares benefited from information, while persistence of punished behavior in Compulsives came at cost.

### Experiment 2: The Impact of Contingency Information Depends on Contingency Strength.

**Fig. 2.**
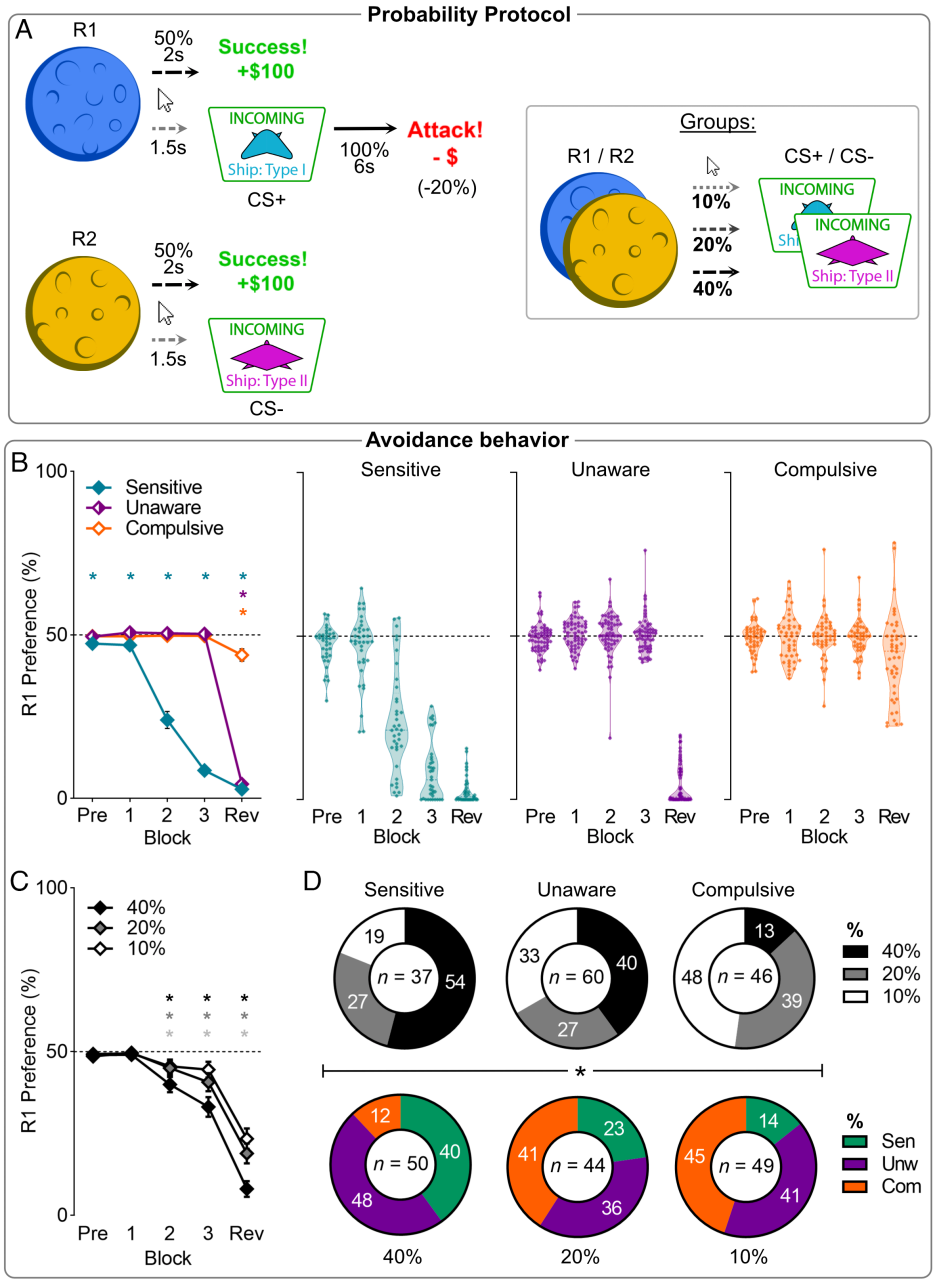
Phenotypes of punishment avoidance across Response-CS probabilities in experiment 2. (*A*) Conditioned punishment task where the probability of a response yielding a CS was 10%, 20%, or 40%. (*B*) Mean (±SEM) punished action preference for the 3 behavioral phenotypes. Pre = pre-punishment; Rev = post-reveal. (*C*) Mean (±SEM) punished action preference across blocks per probability group. Stronger Response-CS probabilities led to greater R1 avoidance. (*D*) Composition of clusters by probability groups (*Top*), and vice versa (*Bottom*). Stronger contingencies drove individuals towards being sensitive, whereas weaker contingencies drove individuals to being Compulsive. *P < .05 single mean *t* test vs. 0.5 preference.

Contingency information is a powerful tool for promoting learning (27–29), so the failure of Compulsive participants to change their behavior after explicit information about why they were being punished is surprising. The visibility of punishment as manipulated by action–punishment contiguity did not contribute to these differences in sensitivity, so in a second experiment [N = 143, n = 110 identifying as female, 17 to 58 y old (*M* = 21.56)], we asked whether visibility could be manipulated in a different way by varying the action–punisher contingency (25). We randomly assigned participants to different punishment probability groups so that they had experience with strong [40% (n = 50)], modest [20% (n = 44)], or weak [10% (n = 49)] response–punishment contingencies (Fig. 2*A*).

Exposure to punishment again reduced punished actions relative to the unpunished actions across blocks [block (linear): F(1,142) = 304.3, *P* < 0.001], with significant avoidance from block P2 onward [P1: t(142) = 1.054, *P* = 0.294; P2-Rev: t(142) ≥ 5.217, *P* < 0.001] (Fig. 2*B*). In contrast to the contiguity manipulation in Experiment 1, the probability manipulation here was influential [overall: F(2,140) = 6.696, *P* = 0.002; block*group: F(2,140) = 9.463, *P* < 0.001] (Fig. 2*C*), with participants trained at 40% showing greater avoidance than 20% (*P* = 0.043) and 10% (*P* = 0.002).

**Fig. 3.**
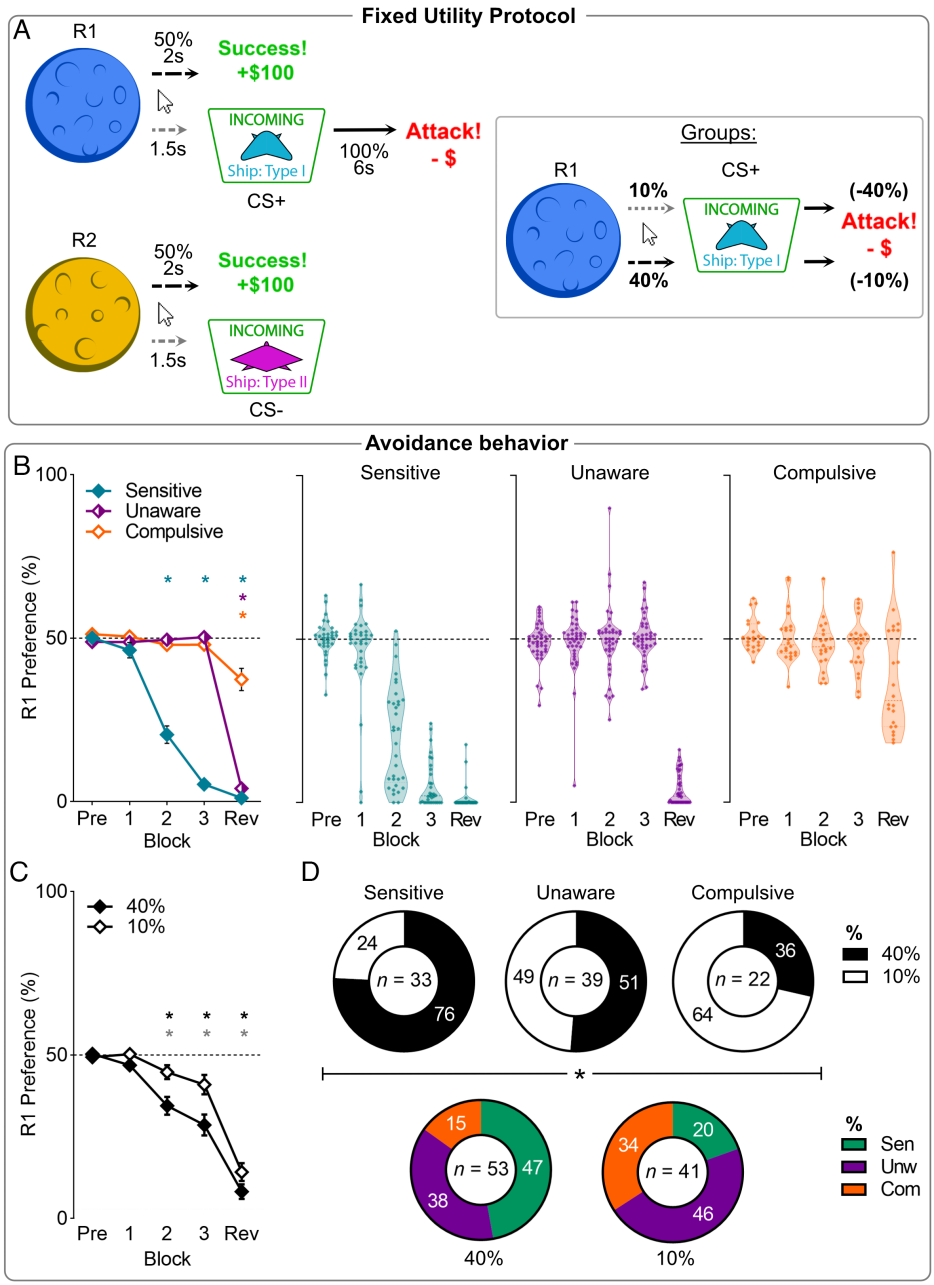
Phenotypes of punishment avoidance under fixed utility in experiment 3. (*A*) Conditioned punishment task with different Response-CS probability conditions (10% vs. 40%), matched on the utility of the punished response. (*B*) Mean (±SEM) punished action preference for the 3 behavioral phenotypes. Pre = pre-punishment; Rev = post-reveal. (*C*) Mean (±SEM) punished action preference across blocks per probability group. Stronger Response-CS probabilities led to greater R1 avoidance. (*D*) Composition of clusters by probability groups (*Top*), and vice versa (*Bottom*). Stronger contingencies drove individuals toward being sensitive, whereas weaker contingencies drove individuals to being compulsive. *P < 0.05 single mean *t* test vs. 0.5 preference.

TwoStep clustering identified the same 3 clusters as previously (Fig. 3*A*) with similar proportions of sensitive (n = 37), unaware (n = 60), and compulsive (n = 46) participants. Clusters did not differ in pre-punishment preference [F(2,140) = 2.847, *P* = 0.061]. Again, participants developed a cluster-dependent preference for the unpunished action across pre-reveal punishment [block: F(1,140) = 291.54, *P* < 0.001; cluster: F(1,140) = 242.341, *P* < 0.001; block*cluster: F(1,140) = 279.024, *P* < 0.001]. Only Sensitives changed their preference across these blocks [block (Sensitive): F(1, 36) = 334.20, *P* < 0.001; (Unaware): F(1,59) = 0.615, *P* = 0.436; (Compulsive): F(1,45) = 0.025, *P* = 0.874]. Again, the contingency reveal increased preference for the unpunished action in a cluster-dependent manner [block: F(1,140) = 461.926, *P* < 0.001; block*cluster: F(1,140) = 254.935, *P* < 0.001]. Compulsives showed significantly less avoidance of the punished relative to unpunished action after the reveal compared to Unawares and Sensitives (*P* < 0.001), who did not differ from each other (*P* = 0.766).

Critically, punishment probability determined cluster phenotypes [χ^2^(4) = 17.18, *P* = 0.002] (Fig. 2*D*). Participants were more likely to be compulsive at weaker rather than stronger punishment contingencies. Moreover, contingency group effects on avoidance depended on cluster. There was no effect of probability group on punishment avoidance if both cluster and probability group were included as between-subjects factors in an ANOVA [group main: F(2,134) = 0.015, *P* = 0.985; group*cluster [F(2,134) = 0.203, *P* = 0.936; block*cluster*group: F(4,134) = 0.857, *P* = 0.492]. Sex of the participant was not a significant factor on avoidance phenotype [χ^2^(2) = 1.289, *P* = 0.525].

### Experiment 3: Compulsivity under Fixed Utility.

These findings show that although explicit information about the causes of punishment remedies punishment insensitivity in some people, the effectiveness of this information depends on the punishment contingency they experienced. However, the probability manipulation confounded changes in contingency visibility with changes in action utility. That is, high probability punishment was more visible but also yielded more point loss, potentially biasing learning on the basis of value. Here, we sought to examine the influence of probability while holding utility constant. We randomly assigned participants [N = 94, n = 66 identifying as female, 2 as other, 18 to 30 y old (*M* = 19.6)] to low-probability severe punishment [10% CS probability, 40% point loss per attack (n = 41)] or high-probability mild punishment [40% CS probability, 10% point loss per attack (n = 53)] (Fig. 3*A*), thereby matching utility of punished actions across the different probability groups. The procedures were otherwise the same as previously.

Punishment again reduced punished relative to unpunished actions [block (linear): F(1,88) = 967.5, *P* < 0.001], with significant avoidance from block P2 onward [P1: t(93) = 1.519, *P* = 0.132; P2-Rev: t(88) = 5.906, *P* < 0.001]. Once again, the probability manipulation was influential [group: F(1,92) = 8.970, *P* = 0.004; block*group: F(1,92) = 5.625, *P* = 0.020] (Fig. 3*C*), with participants punished at 40% showing greater avoidance than those punished at 10% despite the greater severity of punishment in the latter condition.

TwoStep clustering identified the same 3 clusters as previously (Fig. 3*B*)—Sensitive (n = 33), Unaware (n = 39), Compulsive (n = 22). Clusters had similar pre-punishment preferences [F(2,91) = 1.209, *P* = 0.303], but differed during pre-reveal and post-reveal punishment [P1: F(2,91) = 1.078, *P* = 0.345; P2: F(2,91) = 59.39, *P* < 0.001; P3: F(2,91) = 393.4, *P* < 0.001; Rev: F(2,91) = 136.2, *P* < 0.001]. Pre-reveal, Sensitives showed more avoidance of the punished action compared to Unawares and Compulsives (P2-3: *P* < 0.001). Post-reveal, Sensitives and Unawares showed more avoidance than Compulsives (*P* < 0.001). Again, punishment probability dictated cluster phenotypes [χ^2^(2) = 9.035, *P* = 0.011] (Fig. 3*D*). Participants were more likely to be compulsive if the punishment contingency they experienced was weak. Clusters again accounted for group effects on avoidance as there was no probability effect on punishment avoidance when applying both cluster and probability group as between-subjects factors in an ANOVA [group main: F(1,88) = 0.300, *P* = 0.585; group*cluster [F(2,88) = 1.822, *P* = 0.168; block*cluster*group: F(2,88) = 0.314, *P* = 0.732]. Sex of the participant was not a significant factor on avoidance phenotype [χ^2^(4) = 2.858, *P* = 0.582].

### The Nature of Punishment Insensitivity.

In three studies, we identified three phenotypes of punishment avoidance: sensitive participants who exhibited pronounced avoidance through experience alone, Unaware participants who exhibited pronounced avoidance only after being provided contingency information, and compulsive participants who failed to show avoidance following experience or information. To address causes for these differences, we assessed task engagement, self-reported valuations, causal inferences, and trait measures.

**Fig. 4.**
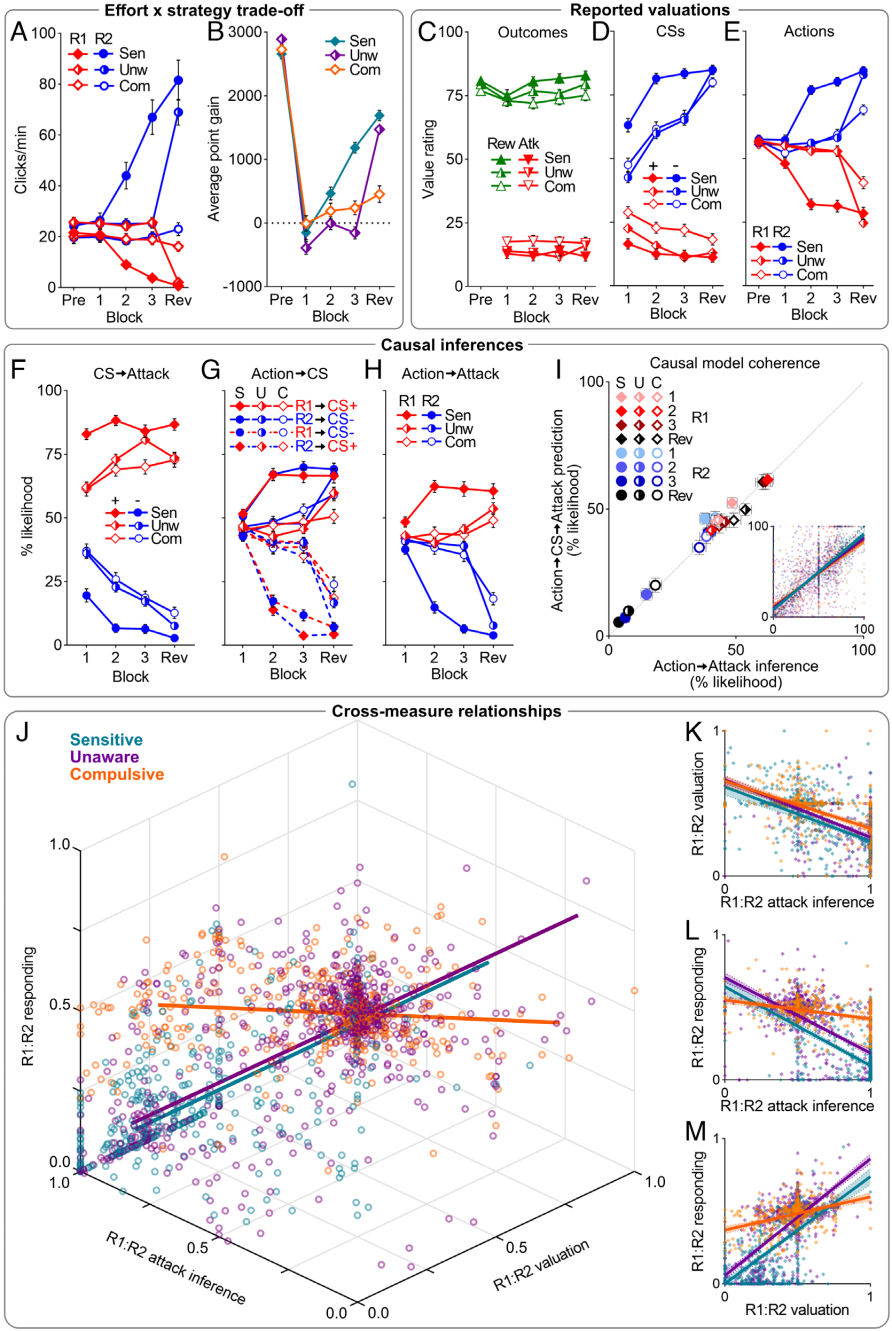
Underpinnings of punishment avoidance phenotypes pooled from experiments 1 to 3. (*A*) Click rates per action during non-CS periods across clusters [Sensitive (Sen), Unaware (Unw), Compulsive (Com)]. (*B*) Average point gain across clusters. (*C*–*E*) Value ratings for point outcomes [i.e., reward (Rew), attack (Atk)] (*C*), CSs (CS+, CS−) (*D*), and actions (R1, R2) (*E*). (*F*–*H*) Self-reported CS→Attack (*F*) and Action→CS (*G*) knowledge, and Action→Attack (*H*) causal inferences. (*I*) Action→Attack inferences per action (R1, R2), per block, against predicted attack likelihood based on Action→CS and CS→Attack knowledge (Action→CS→Attack prediction). Inset: individual Action→Attack inferences by Action→CS→Attack predictions with regression lines per cluster (Sensitive = turquoise, Unaware = purple, Compulsive = orange). (*J*) Relationships between R1:R2 bias in attack inferences, valuations, and behavior (dots: individual datapoints per block across experiments; lines: cluster regression line). (*K*) Relationship between R1:R2 bias in attack inferences and valuations; R1 was increasingly disliked relative to R2 as attack was attributed to R1 over R2, regardless of cluster. (*L*) Relationship between R1:R2 bias in attack inferences and behavior; avoidance of R1 over R2 corresponded to attributions of attack to R1 over R2 for Sensitives and Unawares, but not Compulsives. (*M*) Relationship between R1:R2 bias in valuations and behavior; avoidance of R1 over R2 corresponded to valuation of R2 over R1 for Sensitives and Unawares, but not Compulsives. Data in *A*–*I* are means ± SEM.

Differences in punishment sensitivity were not due to differences in task engagement (Fig. 4*A* and *SI Appendix*, Fig. S1). Clusters had similarly high rates of responding (~46.7 clicks/min) before punishment [F(2,401) = 1.788, *P* = 0.169]. Across pre-reveal punishment, Sensitives decreased punished and increased unpunished responding [action*block: F(1,103) = 84.625, *P* < 0.001], whereas Unawares and Compulsives maintained high rates across both responses [block (Unaware): F(1,190) = 0.051, *P* = 0.821; (Compulsive): F(1,108) = 0.330, *P* = 0.567]. Maintaining both responses is more effortful than focusing on a single response, showing that Unawares and Compulsives were expending as much (if not more) effort as Sensitives in the task, despite accruing less reward (Fig. 4*B* and *SI Appendix*, Fig. S1).

Differences in punishment sensitivity were not due to differences in valuation of reward or punishment (Fig. 4*C* and *SI Appendix*, Fig. S2). Furthermore, all clusters disliked the CS+ over CS− (Fig. 4*D* and *SI Appendix*, Fig. S2), reflecting awareness of CS→Attack contingencies (Fig. 4*F* and *SI Appendix*, Fig. S3). So, all clusters were able to appropriately value outcomes and learn about the environmental predictor of point loss.

Instead, differences in punishment sensitivity reflected differences in instrumental Action→CS knowledge (Fig. 4*G* and *SI Appendix*, Fig. S4) and Action→Attack inferences (Fig. 4*H* and *SI Appendix*, Fig. S4). Sensitives correctly attributed attacks to the punished over unpunished action during pre-reveal punishment, whereas Unawares and Compulsives did not [planet*cluster*block: Experiment 1: F(2,164) = 9.782, *P* < 0.001; Experiment 2: F(2,140) = 33.406, *P* < 0.001]; Experiment 3: F(2,91) = 39.52, *P* < 0.001). These differences mirrored differences in action valuation (Fig. 4*E* and *SI Appendix*, Fig. S2). Before punishment, actions were valued similarly. Across pre-reveal, Sensitives learned to value the unpunished over the punished action, whereas Unawares and Compulsives did not [action*cluster*block: F (2,401) = 76.99, *P* < 0.001]. Crucially, whereas information corrected contingency knowledge and drove action revaluation for Unawares, it was less effective for Compulsives [action*cluster*block: F (1,298) = 9.530, *P* = 0.002]. Compulsives continued to incorrectly attribute attacks to the unpunished action [*P* < 0.001 (vs. Unawares and Sensitives)] and misvalue punished and unpunished actions [planet*cluster*block: F (2,401) = 59.88, *P* < 0.001; R1: *P* < 0.001 and R2: *P* < 0.001 (vs. Unawares and Sensitives)]. Yet, regardless of the veracity of these causal mental models, there was strong coherence between causal beliefs (self-reported Response→Attack likelihoods) and what would be predicted from mediating inferences (i.e., self-reported Response→CS and CS→Attack likelihoods) across clusters (Fig. 4*I* and *SI Appendix*, Fig. S5).

To determine the roles of this attenuated casual belief and value updating among Compulsives in their insensitivity to punishment, we examined how punishment knowledge (R1:R2 attack inferences), action valuations (R1:R2 action value), and behavior (R1:R2 responding) related to each other (Fig. 4 *J*–*M* and *SI Appendix*, Fig. S6). All clusters exhibited coherence in their cognitive and motivational appraisals of actions because punishment knowledge predicted action valuations, regardless of cluster [F(1,1497) = 763.44, *P* < 0.0001, r^2^ = 0.337; cluster: F(2,1493) = 1.83, *P* = 0.161] (Fig. 4*K*). However, clusters differed in how effectively these appraisals translated into behavioral preference. Punishment knowledge generally predicted avoidance [F(1,1499) = 943.0, *P* < 0.0001, r^2^ = 0.386], but this was significantly attenuated in Compulsives [vs. Sensitive: F(1,788) = 117.3, *P* < 0.0001; vs Unaware: F(1,1105) = 113.8, *P* < 0.0001] (Fig. 4*L*). Likewise, action selection was proportional to action value [F(1,1900) = 1352.3, *P* < 0.0001, r^2^ = 0.416) except in Compulsives (F(2,1896) = 66.24, *P* < 0.0001] (vs. Sensitive: F(1,999) = 93.69, *P* < 0.0001; vs. Unaware: F(1,1402) = 154.1, *P* < 0.0001] (Fig. 4*M*). So, on average, Compulsives were impaired in updating their instrumental beliefs and valuations. However, many Compulsives updated their beliefs and values yet still failed to change their behavior.

### Predicting Compulsivity.

Finally, we asked whether we could predict whether an individual was going to become compulsive. Pooling data from unaware (n = 107) and compulsive (n = 59) participants that had received the 20% punishment contingency, we confirmed that post-reveal punished action preference perfectly identified intra-experiment defined clusters in a logistic regression [model χ^2^(1) = 216.92, *P* < 0.001, Nagelkerke r^2^ = 1.0], verifying consistency of clustering across the experiments. Interestingly, pre-reveal punished action preference could not predict cluster identify (*P* = 0.319), highlighting the behavioral similarity of unaware and compulsive phenotypes prior to instruction.

Next, we allowed pre-reveal variables [point gain, reward and attack valuations, action valuation bias, trait subscale scores (*SI Appendix*, Fig. S7)], to conditionally enter into a logistic regression model. Only point gain in the final pre-reveal block entered the model. Unawares tended to lose points in the final pre-reveal block (mean = −315.15 points, SEM = 143.98), whereas Compulsives tended to gain points (mean = 272.46 points, SEM = 166.234) [F(1,165) = 6.506, *P* = 0.012]. However, this difference in point gain could only account for 5.3% of insensitive phenotypes [model χ^2^(1) = 6.587, *P* = 0.010, Nagelkerke r^2^ = 0.053].

## Discussion

We show that people are prone to learning different things about the consequences of their actions and these differences in learning can give rise to compulsive, punishment-resistant behavior. A major source of individual differences is differential acquisition of beliefs regarding the negative consequences of actions. Some people acquire accurate causal beliefs through experience, which they use to avoid punishment while obtaining rewards (punishment-sensitive phenotype). Others form incorrect, albeit internally coherent, causal beliefs based on their experience, leading them to incur punishment they do not like. The possession of incorrect causal beliefs is not entirely problematic because many individuals benefited from explicit contingency information. These individuals (unaware phenotype) responded to a simple information intervention about the causes of punishment that was sufficient to correct their cognitive and motivational appraisals of actions, translating into more optimal behavioral preference. However, some people persisted in detrimental punished behavior despite experience and information intervention (compulsive phenotype).

These findings identify a cognitive pathway to persisting in behavior despite adverse consequences that is predicated on incorrect knowledge and beliefs that individuals acquire about their behavior. The three phenotypes similarly appraised reward and punishment, showing that value distortions did not drive differences in the persistence of behavior here. Moreover, participants engaged in effortful and deliberative cognitive strategies to earn reward and avoid punishment. They formed declarative, internally coherent, mental models of how their actions caused reward and punishment, rather than acting autonomously or habitually relying on stimulus–response procedural knowledge. Of course, value distortions and autonomous behavior can drive insensitivity under some conditions, they just did not appear to be important here. The strong relationship we show between instrumental avoidance and correct awareness is robust (4, 30); even in the Iowa Gambling Task, incorrect beliefs about the avoidability of negative outcomes may be a substantial cause for poor avoidance (31). However, as the unaware phenotype show, incorrect knowledge about the consequences of behavior is not problematic if it can be corrected by information. Our key finding is that the persistence of punished behavior in the compulsive phenotype was due specifically to a failure to incorporate veridical, informational evidence to update incorrect causal beliefs about the consequences and values of actions, as well as a decreased propensity to change behavior if those causal beliefs were updated.

A key condition for this cognitive pathway to punishment insensitivity was infrequent punishment. Punishment contingency manipulations most strongly affected whether individuals were sensitive and compulsive, not unaware. That is, contingency strength dictated whether an individual developed punishment knowledge in the first place and whether additional information could later change established beliefs and behavior. This shows that the impact of information on punishment behaviors and beliefs is moderated not just by its veracity but also by the individual’s experiences (32–34). Incorrect causal beliefs formed under strong punishment contingencies were more sensitive to information-driven updating. In contrast, incorrect causal beliefs formed under weak punishment contingencies were less so, driving individuals toward compulsivity.

Weak contingencies acted like a punishment trap, inoculating individuals against counterevidence that otherwise drove beneficial cognitive and behavioral updating. The mechanisms underlying this punishment trap will be of some interest to isolate. For example, compulsive participants did modestly gain points under punishment. So, they may have persisted in suboptimal behavior, in part, because they did not account for the rewards they were forgoing or because punishment itself served as a discriminative stimulus for further reward (35). In addition, compulsive participants may have been more prone to a confirmation bias (36), devaluing explicit contingency information because it was not consistent with their own beliefs about the task.

Much remains to be learned about this cognitive pathway to punishment insensitivity. None of the self-report measures [covering state negativity, impulsivity, behavioral inhibition/activation, locus of control, and 5-factor personality (*SI Appendix*, Fig. S7)] were reliably associated with any phenotype. Whether other trait constructs, such as cognitive flexibility and intelligence, relate to sensitivity phenotypes will be important to determine. Sex was also unrelated to any phenotype. Men are often overrepresented in problematic behaviors linked to punishment insensitivity and studies in nonhuman animals have identified sex as a variable relevant to individual differences in punishment sensitivity (37). However, in humans, study of these sex differences often rests on the same self-report measures of states and traits (38) that we show do not predict actual differences in punishment learning. It is possible that sex differences affect other pathways to punishment insensitivity (motivational, behavioral) more strongly than they do the cognitive pathway described here. Finally, it is worth noting that insensitive phenotypes (unaware, compulsive) formed the majority of participants across experiments. As stated above, a key factor determining punishment sensitivity is strength of the punishment contingency; it follows that further increasing the consistency of punishment will correspondingly increase the prevalence of sensitive individuals.

Despite the important role that learning from adverse consequences serves in protecting us and sustaining group cooperation as well as social cohesion, actual adverse consequences from risky behaviors are often rare. The probability that any individual risky action such as speeding, social deception, or substance use will have detectable negative consequences is low. Our findings show that when the costs of actions are rare, learning via experience or information does not always yield veridical causal knowledge or optimum decision-making, even if those costs are severe.

## Materials and Methods

### Participants.

Psychology students from University of New South Wales (UNSW) and Western Sydney University (WSU) were recruited in exchange for partial course credit. The experiment was approved by UNSW Human Research Ethics Advisory Panel C (HREAP-C #3385) and WSU Human Research Ethics Committee (HREC #H12809). Prior to commencing the experiment, participants were presented with information about the experiment, the type of data that would be collected, the ethical review process through which the experiment had been evaluated, and how their data would be used and stored. To indicate their consent, participants clicked a button indicating their consent which initiated the experiment. If participants did not consent, participants were told to close their web browser.

Two criteria were used to exclude participants not appropriately engaging in the study: participants were expected to take between 1 and 30 s to answer each question in post-block self-report measures (averaged per page), and participants had to correctly answer two catch questions embedded within questionnaires at the end of the study. For experiment 1, 167 [all UNSW (123 identifying as female, 1 other)] of 263 participants met the inclusion criteria. For experiment 2, 143 [41 UNSW (28 identifying as female), 102 WSU (82 identifying as female)] of 297 participants met the inclusion criteria. For experiment 3, 94 [all UNSW (66 identifying as female, 2 other)] of 215 participants met the inclusion criteria.

### Apparatus and Stimuli.

The experiment was programmed using the jsPsych library (39) and conducted online via the SONA platform. The experiment was programmed to apply full-screen mode to the browser window. The experiment code and stimuli can be found at https://github.com/jessica-c-lee/planets-task/ and https://osf.io/ykun2/, respectively.

#### Game interface.

During game blocks, participants had mouse control of a custom pointer that turned dark when clicking (visual feedback). Two planets [orange, blue (left/right counterbalanced)] were continuously displayed center-left and center-right of the screen. The identity of the punished and unpunished planets (left/right) was randomized. A green ring appeared around a planet whenever the mouse pointer hovered over it (visual feedback). Trade signal (reward countdown) was displayed directly beneath each planet, while reward outcomes were displayed directly above each planet. Accumulated points were continuously displayed top-center of the screen. “Incoming ship” icons [Type I (turquoise), Type II (purple)] were presented in the upper-middle part of the screen. A countdown timer to ship “encounter” was copresented immediately below the ship icon. Ship outcomes (attack, attack deflected, nothing) were presented center-screen, below the encounter countdown. The shield indicator/button was displayed in the lower-middle part of the screen.

#### Post-block self-report assay.

For value ratings, icon and descriptor for task elements (planets, ships, outcomes) were each displayed over a slider (0-100). For causal inferences, each antecedent (R1, R2, Ship I, Ship II) was assayed on a separate page. The antecedent icon was displayed at the top of the screen, and icons for potential consequences (e.g., ships, outcomes) were displayed over 2 sliders each [inference (% likelihood), confidence; both 0-100].

### Procedure.

Participants were randomly assigned to Response-CS contiguity (experiment 1) or probability (experiments 2-3) groups.

#### Initial instructions.

At the beginning of each experiment, participants were told that they would be playing a game over several blocks and that their goal was to gain as many points as possible. They were told they could earn points by “trading” with planets by clicking on them. They were told that additional monetary prizes (unspecified amount) would be awarded to high scorers (unspecified proportion). Following this, they were given a brief multiple-choice comprehension test. Participants had to answer all questions correctly to continue, or else they were returned to the instructions.

#### Pre-punishment phase.

Pre-punishment phase consisted of 2 blocks followed by post-block checks. Each game block lasted 3 min (after which trading was suspended, but any remaining cues/outcomes were presented to completion). Responses on either planet [R1 or R2 (left/right counterbalanced)] initiated a 2-s trading signal (countdown), which had a 50% probability of resulting in signaled reward (“Success! +100”) or nonreward. R1 and R2 countdowns/rewards were independent of each other, such that both planets could be on countdown. During this phase, point gain was maximized by continuous, alternating clicking on both planets, maintaining each on countdown to reward as much as possible.

After each block, values and inferences were assayed. For value, participants were asked on a single page how they felt about reward and planets [0-100 sliders (very negative—neutral—very positive)]. For inferences, they were asked to estimate how often interacting with a planet (one page per planet) would lead to reward [0-100 sliders (never (0%)—sometimes—every time (100%))] and how confident they were about this estimate [0-100 sliders (very uncertain—somewhat uncertain—somewhat confident—very confident)]. Participants had unlimited time to make their responses and could click on a “Continue” button at the bottom of the screen once they had made their ratings. The default slider position was set to 50 (scale midpoint).

#### Punishment phase (pre-reveal vs. post-reveal).

After pre-punishment, participants were given additional instructions warning of local pirates stealing from traders. Participants were informed that their ship has a shield they can activate to prevent theft, but that it will not always be available. They are also reminded the goal is to have as many points as possible. No information about the contingencies between responding and ships, or ships and their outcomes, was provided at this point.

Participants then received 3 pre-reveal punishment blocks. Like pre-punishment, punishment blocks lasted 3 min (plus allowance for cue/outcome termination) and R1/R2 responses were independently and equally rewarded with 50% probability. In addition to reward contingencies, responses triggered incoming ship icons [CS+, CS− (Type I or II ship, counterbalanced)]. R1 exclusively yielded CS+, whereas R2 exclusively yielded CS−. Only one CS could be triggered at a time. CS+ precipitated attacks (6 s following CS+ onset), displayed via an image file with red “Attack! -$” text. The CS− had no negative consequence, as indicated via the message “Ship passed by without incident” in green text. During CS presentations, participants could still make R1/R2 responses and earn rewards unless a shield was active (see below).

At CS onset, a shield charging icon appeared; after 3 s, the icon either informed the participant that the shield was unavailable or became an ACTIVATE button (50% probability of either). If the ACTIVATE button was pressed, the button indicated the shield was active and that 50 points had been deducted. An active shield prevented point loss (“attack deflected” feedback) for that CS trial, but also prevented further trading for the remaining duration of the ship (not cued). Given our focus on preemptive R1 avoidance, the rarity of available shields, and various issues in analyzing CS−related behaviors (*SI Appendix*, Fig. S8), we do not report or discuss shield-related behavior in the text above. Nonetheless, shield use across experiments is reported in *SI Appendix*, Fig. S8.

The default Response-CS parameters across experiments were 20% probability of CS following a response, with 1.5 s delay between the response and CS onset, while attacks caused an immediate −20% loss of accumulated points. Individual parameters were manipulated between-subjects, depending on assigned group. For experiment 1, Response-CS delay was 0 s, 1.5 s, or 3 s (all other parameters default). For experiment 2, Response-CS probability was 10%, 20%, or 40% (all other parameters default). For experiment 3, Response-CS probability was 10% and attack caused −40% point loss, or Response-CS probability was 40% and attack caused −10% point loss (all other parameters default).

Following each punishment block, value and inference were assayed. For value, participants were asked how they felt about reward, planets, ships, and attack. For inferences, they were asked to estimate how often interacting with each planet would lead to reward, Ship Type I, Ship Type II, and attack, and how often Ship Type I and Ship Type II led to attack.

After 3 punishment blocks (with post-block self-reports), participants were given “intel” revealing task contingencies (R1→CS+→Attack, R2→CS−) using both text and figures *SI Appendix*. Text read: “Your signals to the *[blue/orange]* planet [(*left/right)* side] have been attracting pirate ships [Ship: *(Type 1/2)*], that have been stealing your points!” and “Your signals to the *[blue/orange]* planet [*(left/right)*] side have only been attracting friendly ships [Ship: *(Type 1/2)*].” Beneath each of these lines was a diagram of relevant planet, ship, and attack icons, with arrows showing the causal relationship between them.

Participants were then given a final post-reveal punishment block and post-block assay. These were identical to pre-reveal punishment.

### Trait Measure Questionnaires.

At the end of the experiment, participants were administered a battery of self-report measures. These included measures for state depression and anxiety (DASS-21 subscales) (40), impulsivity (New Brief BIS-11) (41), valenced locus of control (Attribution of Responsibility) (42), behavioral inhibition/activation scales (New Brief BIS/BAS) (41), and Big 5 personality (Mini-IPIP) (43). Each questionnaire was administered on one page each (set order). Two catch questions were embedded within Attribution of Responsibility (“select the left-most option, strongly disagree, for this question”) and New Brief BIS/BAS (“select three, very true for me, for this question”) questionnaires. Scores on each subscale were determined (accounting for reverse-coded items) and z-score normalized per dataset.

#### Data analysis.

Data were extracted and processed in MATLAB using custom scripts (available at https://github.com/philjrdb/HCP and https://osf.io/ykun2/), and then imported into SPSS 28 for analysis. Cross-block regression analyses (Action-Attack inferences × Action-CS-Attack predictions, cross-measure relationships) were analyzed in GraphPad Prism 9. For follow-up analyses (Fig. 4), data from across experiments were aggregated; all effects from aggregated data were generally observed per experiment (*SI Appendix*, Figs. S1–S8).

Participants that did not meet engagement criteria (1 to 30 s response times for postblock checks, correct catch questions) were excluded from all subsequent analyses (see *Participants*, *Questionnaires*). Given there were no programmed or observed differences between pre-punishment (Pre) blocks, all data from these blocks were collapsed for the sake of further analysis.

#### Task behavior.

Participant behavior during the Planets and Pirates task was assessed via click rates (clicks/min) on punished and unpunished planets (R1 and R2, respectively) during non-CS periods. Combined R1 and R2 rates were used to calculate a self-normalized preference score [(R1 rate/Overall rate)*100] to indicate the proportion of clicks that were R1. A score of 50% indicates equal rates of R1 and R2, i.e., no preference, whereas score of 0 indicates a complete preference for R2 over R1.

Differences in behavior (click rates, preferences) were analyzed using orthogonal contrasts (see *Contrast Analysis* below). Significant avoidance was also determined using one-sample *t* tests of preference against the null value of 50.

#### Self-reported valuation and inferences.

Valuation of outcomes, CSs, and actions (planets), as well as causal inferences between these, were assessed via self-report at the end of each block (see *Procedure* subsection above). Raw value ratings and inferences (% likelihood rating), each ranging from 0-100, were analyzed using orthogonal contrasts (see *Contrast Analysis* below).

#### Contrast analysis.

Behavior and self-report data across blocks were analyzed using within-subject and mixed between-× within-subject ANOVAs (orthogonal contrasts). Where applicable, within-subject contrasts were block (linear), response (R1 vs. R2), CS (CS+ vs. CS−), inference (correct vs incorrect R→CS). Where applicable, cluster (sensitive, unaware, compulsive) and/or experimental group were used as a between-subject factors. Where applicable, follow-up analyses were conducted per cluster, using one-way ANOVA, or post-hoc between-subject comparisons (Sidak correction).

#### Clustering.

An exploratory TwoStep clustering algorithm was used to identify behavioral phenotypes per experiment. Response preference ratios from the last two punishment blocks (final pre-reveal and post-reveal preference) were used as inputs. In each experiment, 3 clusters were autoidentified via Bayesian information criterion as the optimal solution. Cluster identities derived per experiment were retained for aggregate analyses.

Influence of group or sex on behavioral phenotype was assessed via Pearson’s Chi-square test (2 sided).

#### Response→CS→attack prediction.

To assess the coherence of instrumental causal beliefs, self-reported Response→Attack inferences per block were compared against attack predictions based on self-reported Response→CS and CS→Attack inferences. R1→CS→Attack was calculated as the sum (capped at 100%) of:

R1→CS+→Attack estimate = (R1→CS+ % likelihood) × (CS+→Attack % likelihood)
R1→CS–→Attack estimate = (R1→CS– % likelihood) × (CS–→Attack % likelihood)

The same was done for R2→CS→Attack. Linear regression was used to compare Response→Attack inferences and Response→CS→Attack predictions per cluster.

#### Cross-measure relationships.

Bias in R1:R2 attack inferences, valuations, and behavior were calculated using the ratio formula: R1/(R1 + R2). Self-reported Response→Attack inferences, self-reported action value ratings, or non-CS click rates per block were applied in the formula. This produced a score ranging from 0 to 1; 0.5 indicates no difference between R1 and R2 values (i.e., no bias), scores above 0.5 indicate R1 > R2, while scores below 0.5 indicate R2 > R1. Relationships between ratios were evaluated using linear regressions per cluster; follow-up comparisons between clusters were performed if there was a significant effect of cluster on regression slope.

#### Stepwise logistic regression model for predicting compulsivity.

To identify whether insensitivity phenotype (Unaware vs. Compulsive) could be predicted by behavior or self-report variables, stepwise binary logistic regressions (*P*-to-enter ≤ 0.05, *P*-to-remove ≥ 0.1) were performed on aggregated experiment data. The dependent variable was cluster identity (Unaware vs. Compulsive). Predictor variables across separate regressions were: 1) post-reveal preference, 2) pre-reveal preference (block 3), and 3) pre-reveal point gain, reward value ratings, attack value ratings, R1:R2 valuation bias, and trait subscale scores.

## Supplementary Material

Appendix 01 (PDF)

## Data Availability

Anonymized response rates data have been deposited in GitHub (https://osf.io/ykun2/) and OSF (https://osf.io/z5at4/) (44, 45).

## References

[1] R. Boyd, H. Gintis, S. Bowles, Coordinated punishment of defectors sustains cooperation and can proliferate when rare. Science **328**, 617–620 (2010). 20431013 10.1126/science.1183665

[2] E. Fehr, U. Fischbacher, The nature of human altruism. Nature **425**, 785–791 (2003). 14574401 10.1038/nature02043

[3] J. Henrich , Markets, religion, community size, and the evolution of fairness and punishment. Science **327**, 1480–1484 (2010). 20299588 10.1126/science.1182238

[4] P. Jean-Richard-Dit-Bressel , Punishment insensitivity in humans is due to failures in instrumental contingency learning. Elife **10**, e69594 (2021). 34085930 10.7554/eLife.69594 PMC8177883

[5] C. S. Carver, T. L. White, Behavioral inhibition, behavioral activation, and the affective responses to impending reward and punishment: The BIS/BAS scales. J. Personality Soc. Psychol. **67**, 319–333 (1994).

[6] A. P. Association, Diagnostic and Statistical Manual of Mental Disorders (American Psychiatric Publishing, Arlington, VA, ed. 5, 2013).

[7] M. R. Dadds, K. Salmon, Punishment insensitivity and parenting: Temperament and learning as interacting risks for antisocial behavior. Clin. Child Family Psychol. Rev. **6**, 69–86 (2003). 10.1023/a:1023762009877 12836578

[8] R. J. R. Blair , Passive avoidance learning in individuals with psychopathy: Modulation by reward but not by punishment. Pers. Individ. Differ. **37**, 1179–1192 (2004).

[9] A. J. J. Glover, D. E. Nicholson, T. Hemmati, G. A. Bernfeld, V. L. Quinsey, A comparison of predictors of general and violent recidivism among high-risk federal offenders. Crim. Justice Behav. **29**, 235–249 (2002).

[10] L. Hogarth, Addiction is driven by excessive goal-directed drug choice under negative affect: Translational critique of habit and compulsion theory. Neuropsychopharmacology **45**, 720–735 (2020). 31905368 10.1038/s41386-020-0600-8 PMC7265389

[11] L. Hogarth, M. Field, Relative expected value of drugs versus competing rewards underpins vulnerability to and recovery from addiction. Behav. Brain Res. **394**, 112815 (2020). 32707138 10.1016/j.bbr.2020.112815 PMC7495042

[12] M. Field , Recovery from addiction: Behavioral economics and value-based decision making. Psychol. Addictive Behav. **34**, 182–193 (2020). 10.1037/adb0000518 31599604

[13] W. K. Bickel, L. A. Marsch, Toward a behavioral economic understanding of drug dependence: delay discounting processes. Addiction **96**, 73–86 (2001). 11177521 10.1046/j.1360-0443.2001.961736.x

[14] E. A. Jacobs, W. K. Bickel, Modeling drug consumption in the clinic using simulation procedures: Demand for heroin and cigarettes in opioid-dependent outpatients. Exp. Clin. Psychopharmacol. **7**, 412–426 (1999). 10609976 10.1037//1064-1297.7.4.412

[15] M. L. Banks, S. S. Negus, Insights from preclinical choice models on treating drug addiction. Trends Pharmacol. Sci. **38**, 181–194 (2017). 27916279 10.1016/j.tips.2016.11.002 PMC5258826

[16] C. Luscher, T. W. Robbins, B. J. Everitt, The transition to compulsion in addiction. Nat. Rev. Neurosci. **21**, 247–263 (2020). 32231315 10.1038/s41583-020-0289-z PMC7610550

[17] B. J. Everitt, T. W. Robbins, Drug addiction: Updating actions to habits to compulsions ten years on. Annu. Rev. Psychol. **67**, 23–50 (2016). 26253543 10.1146/annurev-psych-122414-033457

[18] D. Belin, A. Belin-Rauscent, J. E. Murray, B. J. Everitt, Addiction: Failure of control over maladaptive incentive habits. Curr. Opin. Neurobiol. **23**, 564–572 (2013), 10.1016/j.conb.2013.01.025. 23452942

[19] H. Klingemann, M. B. Sobell, L. C. Sobell, Continuities and changes in self-change research. Addiction **105**, 1510–1518 (2010). 19919592 10.1111/j.1360-0443.2009.02770.x

[20] H. Klingemann, L. C. Sobell, Promoting Self-Change from Addictive Behaviors: Practical Implications for Policy, Prevention, and Treatment (Springer, New York, 2007).

[21] L. C. Sobell, M. B. Sobell, T. Toneatto, G. I. Leo, What triggers the resolution of alcohol problems without treatment. Alcoholism: Clin. Exp. Res. **17**, 217–234 (1993). 10.1111/j.1530-0277.1993.tb00752.x 8488958

[22] K. E. Smith, Disease and decision. J. Substance Abuse Treatment **142**, 108874 (2022). 10.1016/j.jsat.2022.108874 PMC9562995 36108442

[23] H. Pickard, S. H. Ahmed, "How do you know you have a drug problem? The role of knowledge of negative consequences in explaining drug choice in humans and rats" in Addiction and Choice, N. Heather, G. Segal, Eds. (Oxford University Press, Oxford, 2016), 10.13140/RG.2.1.2859.3767, pp. 29–48.

[24] G. Weidemann, M. Satkunarajah, P. F. Lovibond, I. Think, Therefore eyeblink: The importance of contingency awareness in conditioning. Psychol. Sci. **27**, 467–475 (2016). 26905277 10.1177/0956797615625973 PMC4831030

[25] J. N. Meindl, L. B. Casey, Increasing the suppressive effect of delayed punishers: A review of basic and applied literature. Behav. Interventions **27**, 129–150 (2012).

[26] T. Chiu, D. Fang, J. Chen, Y. Wang, C. Jeris, “Robust and scalable clustering algorithm for mixed type attributes in large database environment” in Proceedings of the 7th ACM SIGKDD International Conference on Knowledge Discovery and Data Mining 2001 (Association for Computing Machinery, New York, 2001), pp. 263–268.

[27] C. J. Mitchell, J. De Houwer, P. F. Lovibond, The propositional nature of human associative learning. Behav. Brain Sci. **32**, 183–198 (2009), discussion 198–246. 19386174 10.1017/S0140525X09000855

[28] P. F. Lovibond, Causal beliefs and conditioned responses: Retrospective revaluation induced by experience and by instruction. J Exp. Psychol. Learn. Memory Cognit. **29**, 97–106 (2003). 12549586

[29] G. Mertens, Y. Boddez, D. Sevenster, I. M. Engelhard, J. De Houwer, A review on the effects of verbal instructions in human fear conditioning: Empirical findings, theoretical considerations, and future directions. Biol. Psychol. **137**, 49–64 (2018). 29990522 10.1016/j.biopsycho.2018.07.002

[30] F. J. Schmauk, Punishment, arousal, and avoidance learning in sociopaths. J. Abnormal Psychol. **76**, 325–335 (1970). 10.1037/h0030398 4395258

[31] A. Bechara, D. Tranel, A. R. Damasio, Characterization of the decision-making deficit of patients with ventromedial prefrontal cortex lesions. Brain **123**, 2189–2212 (2000). 11050020 10.1093/brain/123.11.2189

[32] M. Scheffer, D. Borsboom, S. Nieuwenhuis, F. Westley, Belief traps: Tackling the inertia of harmful beliefs. Proc. Natl. Acad. Sci. U.S.A. **119**, e2203149119 (2022). 35858376 10.1073/pnas.2203149119 PMC9371746

[33] G. Barron, S. Leider, J. Stack, The effect of safe experience on a warnings’ impact: Sex, drugs, and rock-n-roll. Organ. Behav. Hum. Decis. Processes **106**, 125–142 (2008).

[34] L. Weiss-Cohen, E. Konstantinidis, N. Harvey, Timing of descriptions shapes experience-based risky choice. J. Behav. Decis. Making **34**, 66–84 (2020).

[35] W. C. Holz, N. H. Azrin, Discriminative properties of punishment. J. Exp. Analysis Behav. **4**, 225–232 (1961). 10.1901/jeab.1961.4-225 PMC1404077 13715596

[36] R. S. Nickerson, Confirmation bias: A ubiquitous phenomenon in many guises. Rev. General Psychol. **2**, 175–220 (1998).

[37] T. G. Chowdhury , Sex differences in reward- and punishment-guided actions. Cognit. Affective Behav. Neurosci. **19**, 1404–1417 (2019). 10.3758/s13415-019-00736-w PMC6861363 31342271

[38] C. P. Cross, L. T. Copping, A. Campbell, Sex differences in impulsivity: A meta-analysis. Psychol. Bull. **137**, 97–130 (2011). 21219058 10.1037/a0021591

[39] J. R. de Leeuw, jsPsych: A JavaScript library for creating behavioral experiments in a web browser. Behav. Res. Methods **47**, 1–12 (2015). 24683129 10.3758/s13428-014-0458-y

[40] P. F. Lovibond, S. H. Lovibond, The structure of negative emotional states: Comparison of the Depression Anxiety Stress Scales (DASS) with the beck depression and anxiety inventories. Behav. Res. Therapy **33**, 335–343 (1995). 10.1016/0005-7967(94)00075-u 7726811

[41] M. E. Morean , Psychometrically improved, abbreviated versions of three classic measures of impulsivity and self-control. Psycholog. Assess. **26**, 1003–1020 (2014). 10.1037/pas0000003 PMC4152397 24885848

[42] C. R. Brewin, D. A. Shapiro, Beyond locus of control: Attribution of responsibility for positive and negative outcomes. British J. Psychol. **75**, 43–49 (1984).

[43] M. B. Donnellan, F. L. Oswald, B. M. Baird, R. E. Lucas, The mini-IPIP scales: Tiny-yet-effective measures of the Big Five factors of personality. Psychol. Assess. **18**, 192–203 (2006). 16768595 10.1037/1040-3590.18.2.192

[44] J. Lee, Planets Task. Github. https://github.com/jessica-c-lee/planets-task. Deposited 8 September 2020.

[45] P. Jean-Richard-dit-Bressel, HCP2. OSF. https://osf.io/z5at4/. Deposited 10 January 2023.

