# Supplementary material for: A cognitive pathway to punishment insensitivity
Source: Proc Natl Acad Sci U S A. 2023 Apr 3;120(15):e2221634120. doi: 10.1073/pnas.2221634120 (PMC10104546; doi:10.1073/pnas.2221634120)
Supplement: Supplementary file 1 — Appendix 01 (PDF) [file pnas.2221634120.sapp.pdf]

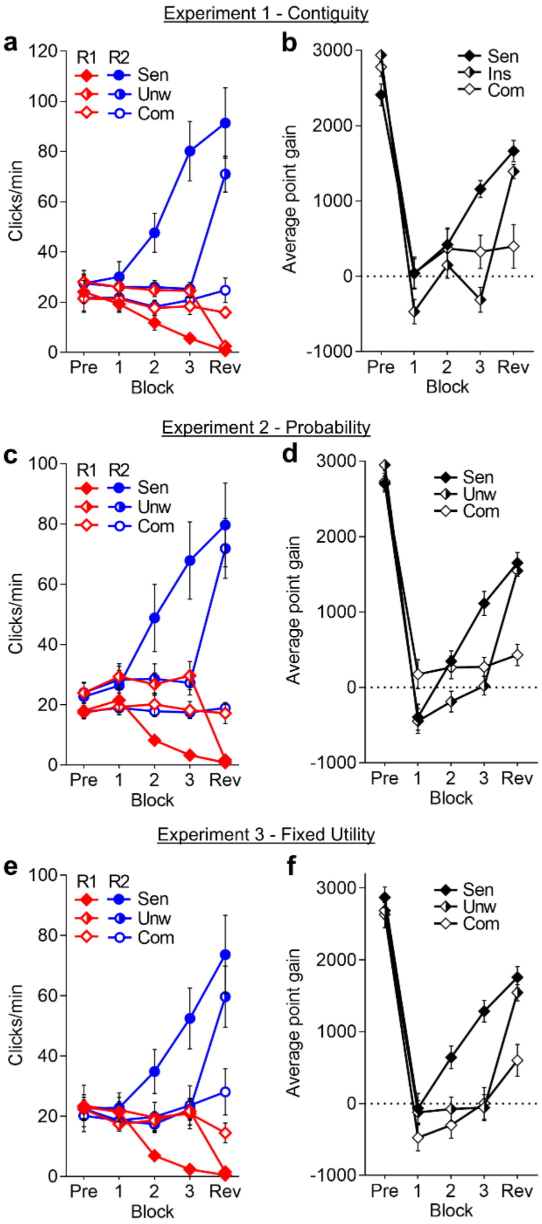

Figure S1. Click rates and point gain per experiment. Mean (±SEM) click rates per action (R1, R2) during non-CS periods across blocks were consistent across Experiments 1 **[A]**, 2 **[C]**, and 3 **[E]**. Clusters did not differ in pre-punishment R1 ([Expt 1]: F(2,164) = .751, p=.474; [Expt 2]: F(2,140) = 1.616, p=.202; [Expt 3]:F(2,91) = .014, p=.987)) or R2 click rates ([Expt 1]: F(2,164) = .579, p=.562; [Expt 2]: F(2,140) = 1.005, p=.369; [Expt 3]: F(2,91) = .085, p=.919). Cluster click rates diverged across pre-reveal punishment (Action*Block*Cluster: [Expt 1]: F(2,164) = 64.854, p<.001; [Expt 2]: F(2,140) = 33.170, p<.001; [Expt 3]: F(2,91) = 18.498, p<.001), driven by gradual

response discrimination in Sensitives but not Unawares or Compulsives. The contingency reveal drove cluster-dependent changes in response allocation (Action*Block*Cluster: [Expt 1]: $F(2,164) = 22.274$, $p<.001$; [Expt 2]: $F(2,140) = 33.170$, $p<.001$; [Expt 3]: $F(2,91) = 18.498$, $p<.001$), most notably in Unawares. In the post-reveal block, Compulsives responded more on R1 ([Expt 1]: $p<.001$; [Expt 2]: $p<.001$; [Expt 3]: $p<.001$) and less on R2 ([Expt 1]: $p<.001$; [Expt 2]: $p<.001$; [Expt 3]: $p=.033$) than Sensitives and Unawares, who did not significantly differ on either response ([Expt 1]: $p\geq.312$; [Expt 2]: $p\geq.922$; [Expt 3]: $p\geq.723$). These differences in responding were consequential, leading to similar cluster differences in point gain across Experiments 1 **[B]**, 2 **[D]**, and 3 **[F]** (mean±SEM). During the final pre-reveal punishment block, Sensitives were gaining the most points (vs Com/Unw [Expt 1]: $p\leq.033$; [Expt 2]: $p<.001$; [Expt 3]: $p<.001$). In the post-reveal block, Compulsives were gaining the least points (vs Sen/Unw [Expt 1]: $p<.001$; [Expt 2]: $p<.001$; [Expt 3]: $p<.001$).

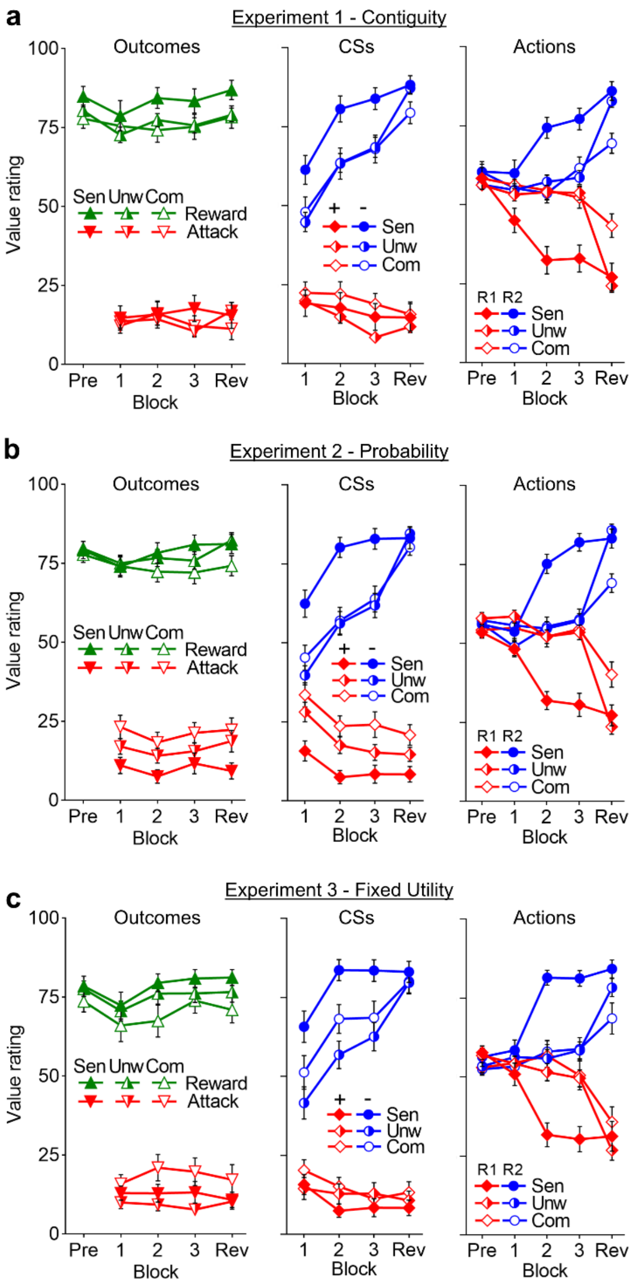

Figure S2. Figure S2. Self-reported valuations per experiment. Mean (±SEM) valuations of outcomes (left), CSs (middle) and actions (right) across Experiments 1 [**A**], 2 [**B**], and 3 [**C**]. **[A,B,C** left] Across experiments and blocks, each cluster reported liking the reward (cluster [Expt 1]: $F(2,164) = 1.943$, $p=.147$; [Expt 2]: $F(2,140)=.979$, $p=.378$; [Expt 3]: $F(2,91)=1.851$, $p=.163$) and disliking attacks (cluster [Expt 1]: $F(2,164) =.275$, $p=.760$; [Expt 2]: $F(2,140) = 4.361$, $p=.015$; [Expt 3]: $F(2,91) = 3.612$, $p=.031$). [**A,B,C** middle] All

clusters developed discriminated CS valuations before the reveal (CS*block [Expt 1]: F(1,164) = 299.99, p<.001; [Expt 2]: F(1,140) = 80.08, p<.001; [Expt 3]: F(1,91) = 34.034, p<.001), although Sensitives had greater pre-reveal discrimination (CS*cluster [Expt 1]: F(2,164) = 3.577, p=.030; [Expt 2]: F(2,140) = 18.055, p<.001; [Expt 3]: F(2,91) = 8.110, p<.001). Following the reveal, cluster were largely similar in their valuations of CS± ([Expt 1]: F(2,164) = .574, p=.564; [Expt 2]: F(2,140)=4.705,p=.011; [Expt 3]: F(2,91)=.759,p=.471) or CS- ([Expt 1]: F(2,164) = 3.179, p=.044; [Expt 2]: F(2,140)=.768,p=.466; [Expt 3]: F(2,91)=.300,p=.742). [**A,B,C** right] There were qualitative differences in how clusters revalued actions (R1, R2) across pre-reveal punishment (action*cluster*block [Expt 1]: F(2,164) = 18.43, p<.001; [Expt 2]: F(2,140) = 33.406, p<.001; [Expt 3]: F(2,91) = 23.213, p<.001). Sensitives (action [Expt 1]: F(1,33) = 67.835, p<.001; [Expt 2]: F(1,36) = 99.73, p<.001; [Expt 3]: F(1,32)=69.30,p<.001), but not Unawares ([Expt 1]: F(1,91) = 2.439, p=.122; [Expt 2]: F(1,59)=.346, p=.558; [Expt 3]: F(1,38)=3.089, p=.087) or Compulsives ([Expt 1]: F(1,40) = 1.602, p=.213; [Expt 2]: F(1,45)=.101, p=.752; [Expt 3]: F(1,21)=.543, p=.469), discriminated action values before the reveal. The reveal caused cluster-dependent revaluation of actions (action*cluster*block [Expt 1]: F(2,164) = 20.728, p<.001; [Expt 2]: F(2,140) = 28.474, p<.001; [Expt 3]: F(2,91) = 12.11, p<.001). All clusters discriminated action values in the post-reveal block, although Compulsives tended to discriminate less than other clusters (action*cluster [Expt 1]: F(2,164) = 14.663, p<.001; [Expt 2]: F(2,140) = 13.57, p<.001; [Expt 3]: F(2,91) = 2.427, p=.094).

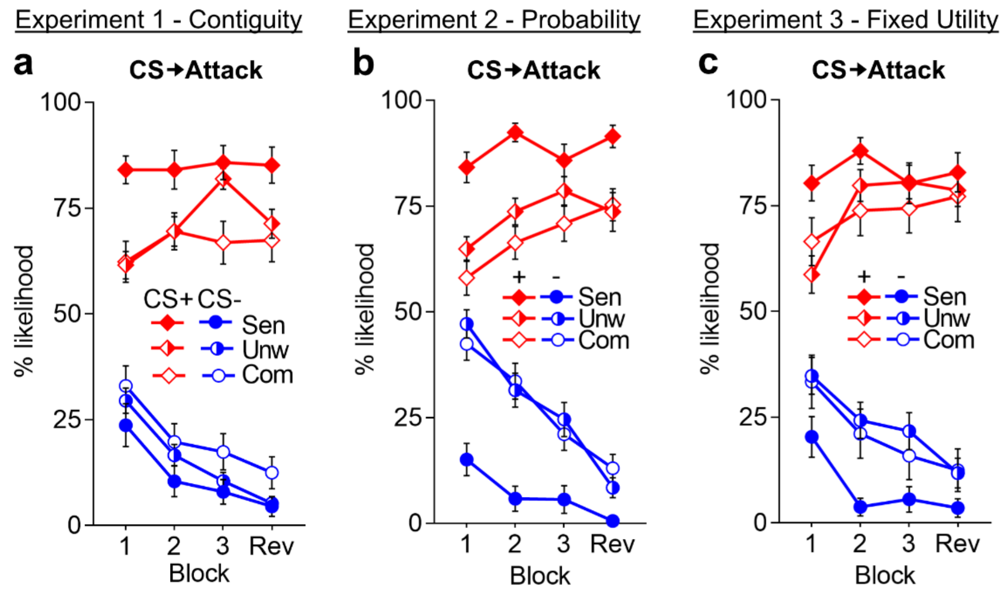

Figure S3. CS→Attack inferences per experiment. Mean (±SEM) CS→Attack inferences for Experiments 1 **[A]**, 2 **[B]**, and 3 **[C]**. Generally, participants attributed attacks to CS± over CS- before the reveal, developing this knowledge across blocks (CS*block [Expt 1]: $F(1,164) = 46.26$, $p<.001$; [Expt 2]: $F(1,140) = 80.08$, $p<.001$; [Expt 3]: $F(1,91) = 20.515$, $p<.001$). Sensitives were typically better at this discrimination (CS*cluster [Expt 1]: $F(2,164) = 6.62$, $p=.002$; [Expt 2]: $F(2,140) = 18.055$, $p<.001$; [Expt 3]: $F(2,91) = 6.421$, $p=.002$), although every cluster discriminated CS values from 1st block of punishment (all $|t| \geq 2.677$, $p \leq .010$). Depending on experiment, the contingency reveal had modest or no significant effect on CS discrimination per cluster (CS*block*cluster [Expt 1]: $F(2,164) = 1.444$, $p=.239$; [Expt 2]: $F(2,140)= 4.633$,$p=.011$; [Expt 3]: $F(2,91) = .09$, $p=.914$). Following the reveal, there were inconsistent cluster differences in attack attributions to CS± ([Expt 1]: $F(2,164) = 3.343$, $p=.038$; [Expt 2]: $F(2,140) = 5.027$, $p=.008$; [Expt 3]: $F(2,91) =.400$, $p=.672$) and CS- ([Expt 1]: $F(2,164) = 2.768$, $p=.066$; [Expt 2]: $F(2,140) =5.282$, $p=.006$; [Expt 3]: $F(2,140) = 2.028$, $p=.137$), with all clusters strongly attributing attacks to CS± and not CS- (all $|t| \geq 7.858$, $p<.001$).

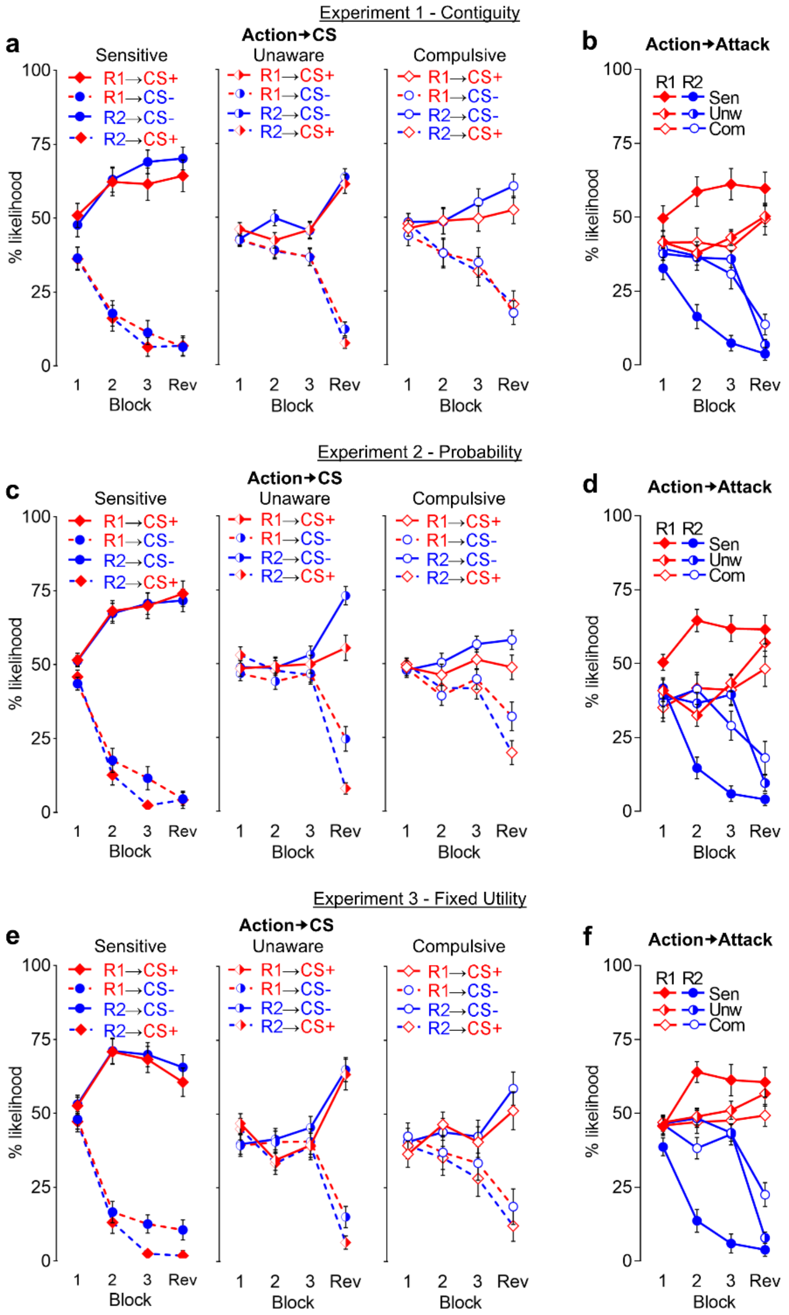

Figure S4. Mean (±SEM) Action→CS inferences per cluster (Sensitive [**left**], Unaware [**middle**], Compulsive [**right**]) for Experiments 1 [**A**], 2 [**C**], and 3 [**E**]. Before the reveal, only Sensitives effectively learned to selectively attribute CS± to R1 and CS- to R2 (correct inferences [solid line]), while discounting incorrect R1→CS- and R2→CS± inferences (dashed lines) (Correct*cluster*block [Expt 1]: $F(2,164) = 10.538$, $p<.001$; [Expt 2]: $F(2,140)=30.07, p<.001$; [Expt 3]: $F(2,91) = 39.52$, $p<.001$). The reveal largely addressed this cluster-dependent failure in instrumental learning (Correct*cluster*block

[Expt 1]: F(2,164) = 10.538, p<.001; [Expt 2]: F(2,140)=30.07,p<.001; [Expt 3]: F(2,91) = 25.68, p<.001), correcting Action→CS attributions for Unawares (Correct*Block [Expt 1]: F(1,91) = 85.80, p<.001; [Expt 2]: F(1,59)=81.60,p<.001; [Expt 3]: F(1,38) = 72.84, p<.001) and Compulsives ([Expt 1]: F(1,40) = 6.497, p=.015; [Expt 2]: F(1,45)=14.84,p<.001; [Expt 3]: F(1,21) = 12.44, p=.002), but not Sensitives ([Expt 1]: F(1,33) = .917, p=.345; [Expt 2]: F(1,36)=1.162,p=.288; [Expt 3]: F(1,32) = 1.573, p=.219). Clusters also differed on their self-reported Action→Attack inferences across Experiments 1 [**B**], 2 [**D**], and 3 [**F**]. Before the reveal, Sensitives correctly attributed attacks to R1 over R2 (Action [Expt 1]: F(1,33) = 85.60, p<.001; [Expt 2]: F(1,36)=146.97,p<.001; [Expt 3]: F(1,32) = 96.75, p<.001), whereas Unawares ([Expt 1]: F(1,91) = 4.601, p=.035; [Expt 2]: F(1,59)= 2.402,p=.127; [Expt 3]: F(1,38) = .040, p=.843) and Compulsives ([Expt 1]: F(1,40) = 1.484, p=.230; [Expt 2]: F(1,45)=3.288,p=.076; [Expt 3]: F(1,21) = .779, p=.387) generally did not. The contingency reveal addressed poor punishment knowledge, increasing discrimination for Unawares (Action*Block [Expt 1]: F(1,91) = 50.05, p<.001; [Expt 2]: F(1,59)=52.33,p<.001; [Expt 3]: F(1,38) = 57.42, p<.001) and Compulsives ([Expt 1]: F(1,40) = 14.99, p<.001; [Expt 2]: F(1,45)=14.85,p<.001; [Expt 3]: F(1,21) = 3.714, p=.068), but not Sensitives ([Expt 1]: F(1,33) = .147, p=.704; [Expt 2]: F(1,36)=.064,p=.802; [Expt 3]: F(1,32) = .104, p=.749).

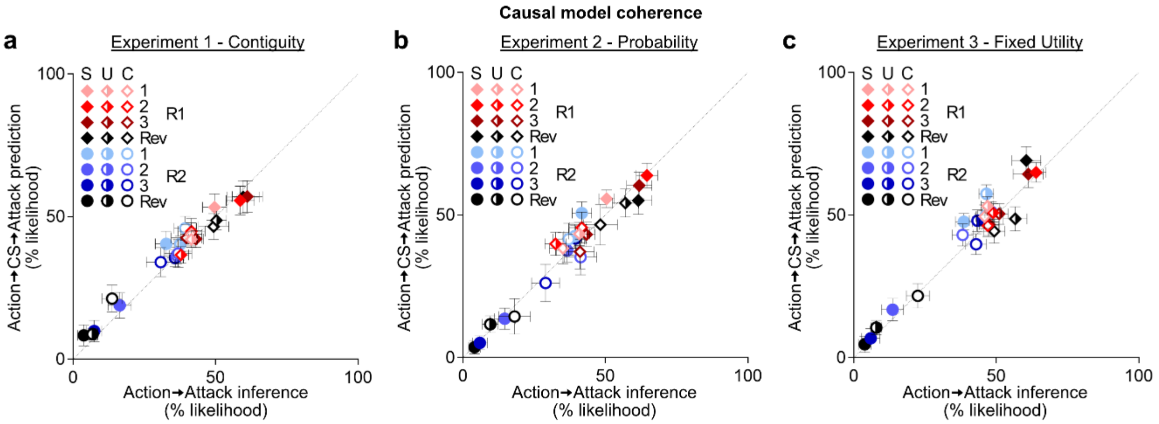

Figure S5. Causal model coherence per experiment. Relationship between self-reported Action-Attack inference and prediction from self-reported Action-CS and CS-Attack inferences (Action-CS-Attack prediction) across actions (R1, R2), punishment blocks (1, 2, 3, Rev) and clusters (Sensitive [S], Unaware [U], Compulsive [C]) for Experiments 1 [**A**], 2[**B**] and 3 [**C**] (Mean±SEM). Across experiments, there was a strong correspondence between Action-Attack inferences and attack predictions derived from mediating inferences, regardless of cluster.

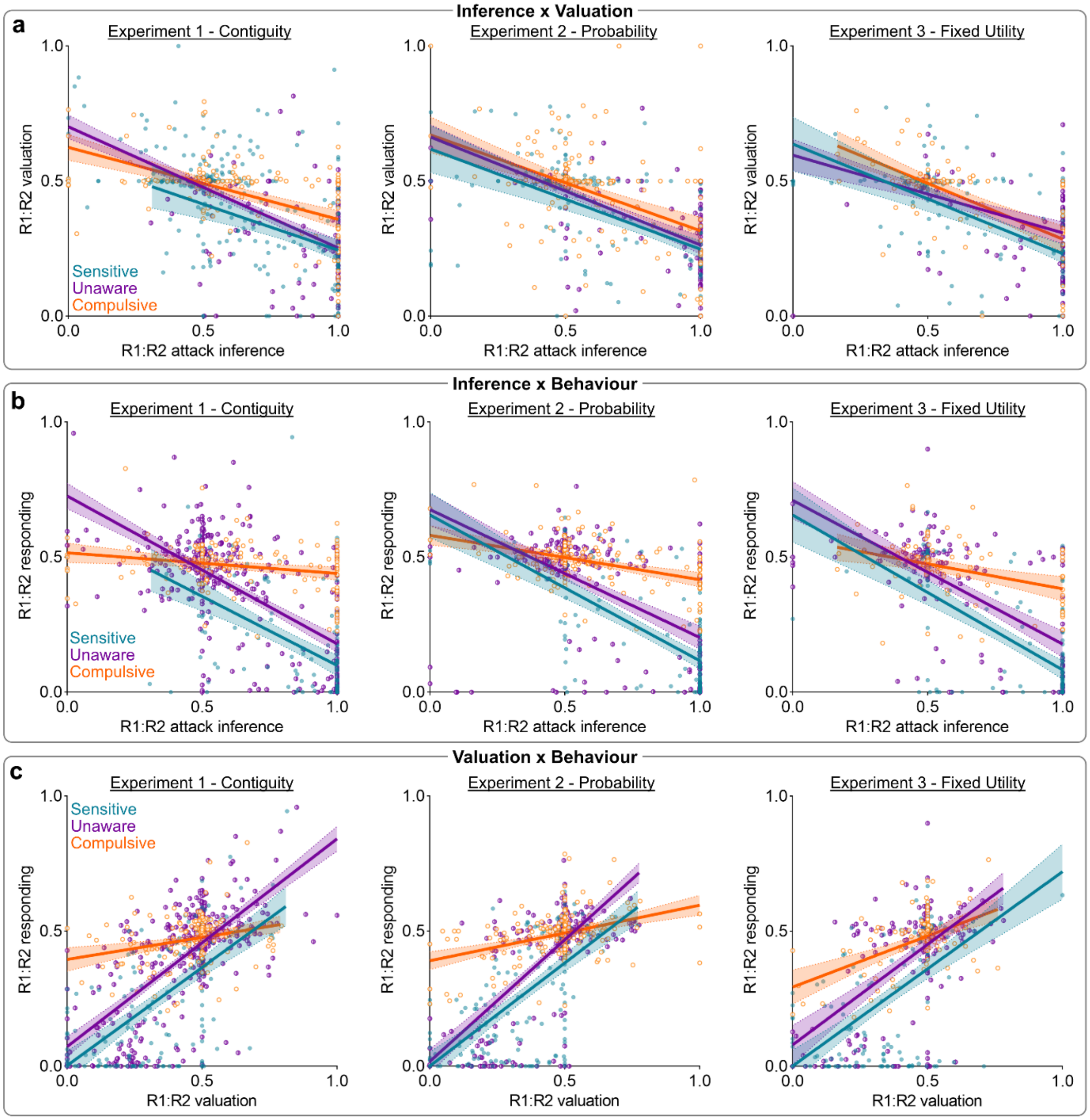

Figure S6. Cross-measure relationships per experiment. [**A**] Relationship between R1:R2 bias in Response→Attack inferences and response valuations across blocks for Experiment 1 (**left**), 2 (**middle**) and 3 (**right**). Generally, the more attack was attributed to R1 over R2, the less R1 was valued relative to R2. Although this relationship depended on cluster for Experiment 1 ($F(2,594) = 5.596$, $p=.0039$), there was no significant effect of cluster for Experiments 2 ($F(2,534) = .265$, $p=.768$) or 3 ($F(2,353) = 1.973$, $p=.141$), nor in the aggregate data ($F(2,1493) = 1.830$, $p=.161$). This cluster difference for Experiment 1 was driven by a slope difference between insensitive

clusters ([Unaware vs Compulsive]: $F(1,469)=12.05$, $p=.0006$); Sensitive did not significantly differ from Unaware ($F(1,450) = 2.173$, $p=.141$) or Compulsive ($F(1,269) = .9698$, $p=.326$) slopes. [**B**] Relationship between R1:R2 bias in Response→Attack inferences and response preference across blocks for Experiment 1 (left), 2 (middle) and 3 (right). Generally, the more attack was attributed to R1 over R2, the less R1 was clicked relative to R2. This relationship depended on cluster for Experiment 1 ($F(2,595) = 37.2$, $p<.0001$), 2 ($F(2,535) = 16.66$, $p<.0001$), and 3 ($F(2,353) = 10.95$,$p<.0001$). This cluster difference was driven a flatter slope for Compulsives relative to Sensitives ([Expt 1]: $F(1,269)=40.37$, $p<.0001$; [Expt 2]: $F(1,305)=44.25$, $p<.0001$; [Expt 3]: $F(1,206)=22.03$, $p<.0001$) and Unawares ([Expt 1]: $F(1,470)=77.42$, $p<.0001$; [Expt 2]: $F(1,398)=23.81$, $p<.0001$; [Expt 3]: $F(1,229)=17.71$, $p<.0001$); Sensitive and Unaware slopes did not significantly differ ([Expt 1]: $F(1,451)=.2195$, $p=.6397$; [Expt 2]: $F(1,367)=.8639$, $p=.353$; [Expt 3]: $F(1,271)=.2649$, $p=.607$). [**C**] Relationship between R1:R2 bias in response valuation and response preference across blocks for Experiment 1 (left), 2 (middle) and 3 (right). Generally, the more R2 was valued over R1, the more R2 was clicked over R1. This relationship depended on cluster for Experiment 1 ($F(2,761) = 25.56$, $p<.0001$), 2 ($F(2,676) = 48.78$, $p<.0001$), and 3 ($F(2,447) = 4.303$,$p=.0141$). This cluster difference was driven a flatter slope for Compulsives relative to Sensitives ([Expt 1]: $F(1,344)=37.53$, $p<.0001$; [Expt 2]: $F(1,386)=57.44$, $p<.0001$; [Expt 3]: $F(1,261)=7.517$, $p=.0065$) and Unawares ([Expt 1]: $F(1,602)=59.19$, $p<.0001$; [Expt 2]: $F(1,502)=115.7$, $p<.0001$; [Expt 3]: $F(1,290)=9.210$, $p=.0026$); Sensitive and Unaware slopes did not significantly differ ([Expt 1]: $F(1,576)=.3210$, $p=.5712$; [Expt 2]: $F(1,464)=2.932$, $p=.086$; [Expt 3]: $F(1,343)=.051$, $p=.822$).

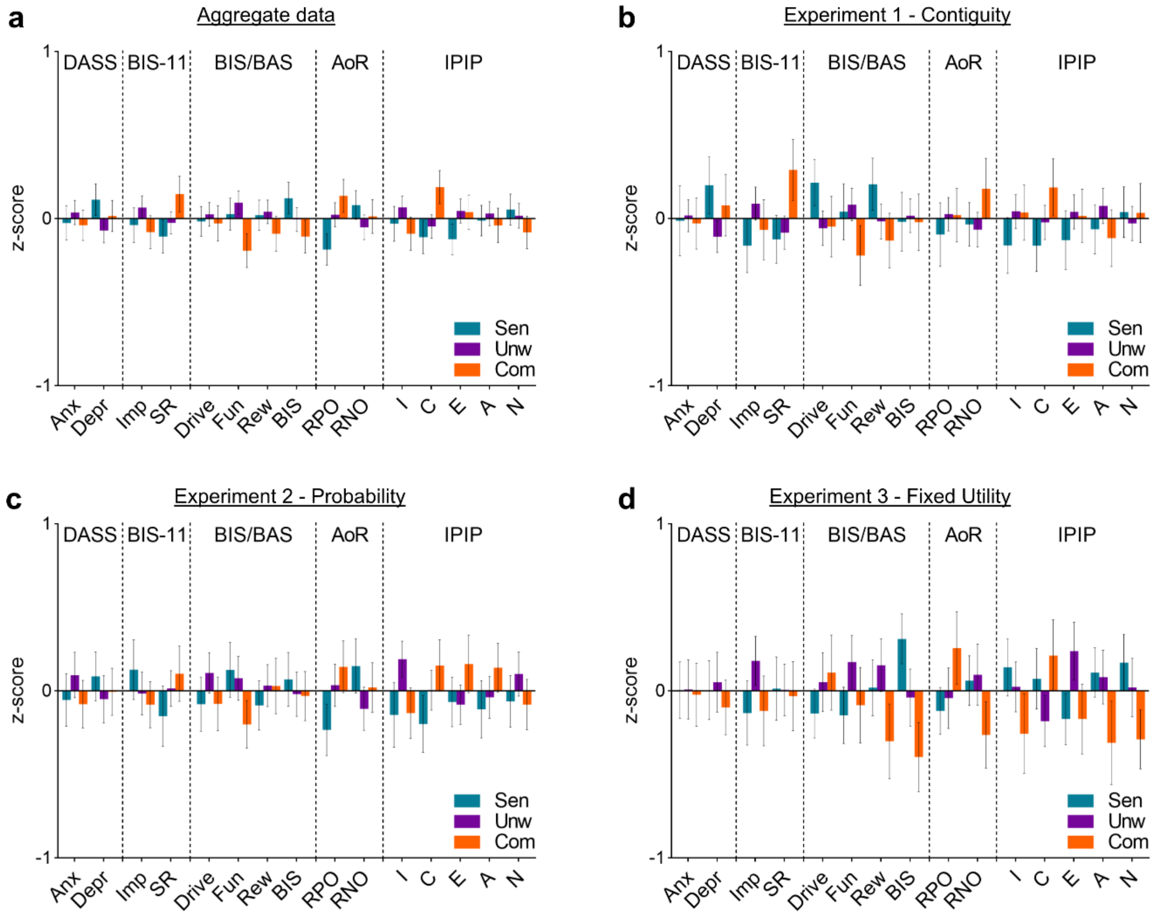

Figure S7. Normalized scores on state/trait measure. Mean (±SEM) normalized subscale scores per cluster on self-report state/trait scales (normalized per dataset) across aggregated data **[A]** and Experiments 1-3 **[B-D]**. Depression Anxiety Stress Scale [DASS21]: Anxiety [Anx], Depression [Dep]; New Brief Barratt Impulsiveness Scale [BIS-11]: Impulsive Behavior [Imp], Poor Self-Regulation [SR]; BIS/BAS: Drive, Fun-seeking [Fun], Reward Responsiveness [Rew], Behavioral Inhibition [BIS]; Attribution of Responsibility [AoR]: Responsibility for Positive Outcomes [RPO], Responsibility for Negative Outcomes [RNO]; Mini-International Personality Item Pool [IPIP]: Intellect/Imagination [I], Conscientiousness [C], Extraversion [E], Agreeableness [A], Neuroticism [N].Clusters did not significantly differ on any subscale in the aggregated data ($F(2,401) \leq 2.950$, $p \geq .053$ **[A]**), Experiment 1 ($F(2,164) \leq 2.371$, $p \geq .097$ **[B]**), or Experiment 2 ($F(2,140) \leq 1.896$, $p \geq .154$ **[C]**). There was a significant cluster difference for the BIS subscale in Experiment 3 ($F(2,91)=3.541$, $p=.033$ **[D]**), driven by significantly lower scores for Compulsives relative to Sensitives ($p=.029$); Unawares did not significantly differ from other clusters ($p \geq .343$). Clusters did not significantly differ on any other subscale in Experiment 3 ($F(2,91) \leq 1.923$, $p \geq .152$).

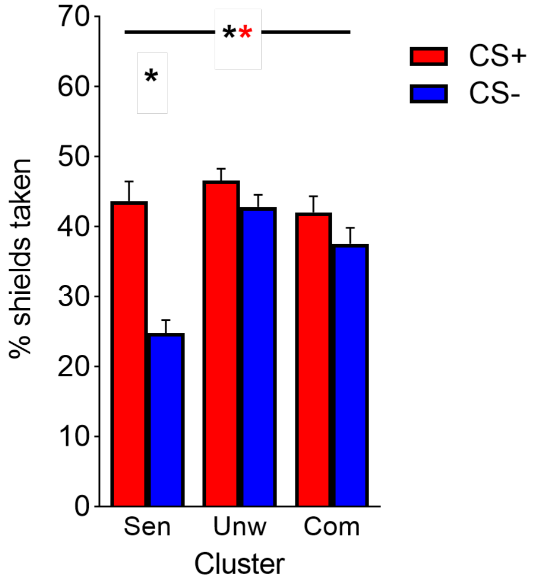

Figure S8. Shield utilization per cluster prior to reveal across experiments. Prior to contingency reveal, participants engaged the shield button (active avoidance response) when available more for the CS+ than CS- (CS: $F(1,395) = 34.51$, $p < .001$). This effect depended on cluster (CS*cluster: $F(2,395)=9.48$, $p < .001$). This was driven by cluster differences in shield use for CS- ($F(2,401) = 21.03$, $p < .001$) over any difference in CS+ shield use ($F(2,395) = 1.28$, $p = .279$). Sensitives used shields during CS- less than Unawares and Compulsives ($p < .001$); Unawares and Compulsives did not differ in CS- shield use ($p = .158$). It is critical to note that this data is aggregated from across blocks due to the rarity of CSs and shield availability in any given block (e.g. no CS+ appearances in later blocks for Sensitives), which undermines the contrast analysis approach used for other measures. Therefore, this data belies an asymmetry in how different blocks contribute to shield use per cluster. This pattern of active avoidance across clusters was observed in the original study (4).
